# Regulation of Photosynthetic Electron Transport and Photoinhibition

**DOI:** 10.2174/1389203715666140327105143

**Published:** 2014-06

**Authors:** Thomas Roach, Anja Krieger Krieger-Liszkay

**Affiliations:** 1 Institut für Botanik, Leopold-Franzens-Universität-Innsbruck, Sternwartestrasse 15, 6020 Innsbruck, Austria;; 2 Commissariat à l’Energie Atomique (CEA) Saclay, iBiTec-S, CNRS UMR 8221, Service de Bioénergétique, Biologie Structurale et Mécanisme, 91191 Gif-sur-Yvette Cedex, France

**Keywords:** Electron transport, light stress, non-photochemical quenching, photoinhibition, photosynthesis, reactive oxygen species, regulation.

## Abstract

Photosynthetic organisms and isolated photosystems are of interest for technical applications. In nature, photosynthetic electron transport has to work efficiently in contrasting environments such as shade and full sunlight at noon. Photosynthetic electron transport is regulated on many levels, starting with the energy transfer processes in antenna and ending with how reducing power is ultimately partitioned. This review starts by explaining how light energy can be dissipated or distributed by the various mechanisms of non-photochemical quenching, including thermal dissipation and state transitions, and how these processes influence photoinhibition of photosystem II (PSII). Furthermore, we will highlight the importance of the various alternative electron transport pathways, including the use of oxygen as the terminal electron acceptor and cyclic flow around photosystem I (PSI), the latter which seem particularly relevant to preventing photoinhibition of photosystem I. The control of excitation pressure in combination with the partitioning of reducing power influences the light-dependent formation of reactive oxygen species in PSII and in PSI, which may be a very important consideration to any artificial photosynthetic system or technical device using photosynthetic organisms.

## INTRODUCTION

In oxygenic photosynthesis light energy, with the help of light-harvesting antenna, is used to drive two specialized complexes called photosystem I (PSI) and photosystem II (PSII). The energy released from a captured photon triggers charge separation in PSI and PSII reaction centres, and subsequent electron transfer reactions, enabling electrons and protons to be taken from H_2_O and the release of O_2_. The released electrons are transported via a series of redox-active co-factors to reduce a final electron acceptor, such as NADP^+^. At the same time a proton gradient (ΔpH) is generated across the thylakoid membrane that provides, together with the electrochemical gradient (ΔΨ), the proton motive force required for the synthesis of ATP. Charge separation creates a positive charge at the donor side of the photosystems, which is reduced by plastocyanin in PSI and by electrons from the water-splitting complex in PSII. While the absorption of light energy by antenna systems is highly efficient (i.e., extinction coefficient of chl: ε: approx. 10^5 ^M^-1 ^cm^-1^), and helped by a broad range in absorption wavelengths by chlorophyll *a*, *b* and a number of carotenoid molecules, energy is lost during charge separation, stabilization and onward electron transfer. In photosynthesis, further energy is lost during CO_2_ fixation, especially under suboptimal conditions. In an optimal environmental setting, the maximum conversion of solar energy to biomass is estimated at 6%, but only for the most efficient plants [1, 2].The reaction centres of PSI and PSII convert photon energy into electrical potentials with very high efficiency (80 ± 15 % and 45 ± 10 %, respectively) [3] when measured on a microsecond timescale, making them highly attractive as potential photovoltaic devices [4, 5]. On longer timescales, however, the energy conversion efficiency is largely reduced to about 40% for PSI [6]. Technical applications are increasingly exploiting the efficiency of photosynthesis for solid-state devices mimicking photovoltaic cells. Photo-electric currents have been achieved with immobilized chloroplasts [7], thylakoid membranes [8-10], PSII [11, 12] or PSI [13-16] core complexes and isolated reaction centres [17-19]. One of the most promising current bio-photovoltaic without using elaborate or expensive surface chemistries is a PSI complex attached to a semiconductor, achieving a photocurrent density of 362 µA/cm^2^ and 0.5 V [15]. Purified complexes [20], photosynthetic membranes [21-24] or whole organisms [25-29] have also been placed on electrodes for assembling biosensors (for review see [30, 31]), mainly for the detection of pollutants, but also as components for future H_2_ production devices [32]. As PSI has a higher efficiency and is less prone to photoinhibion than PSII (see later), it could be more suitable for biomimetic devices (for recent reviews see [32-34]).

**Fig. (1):**
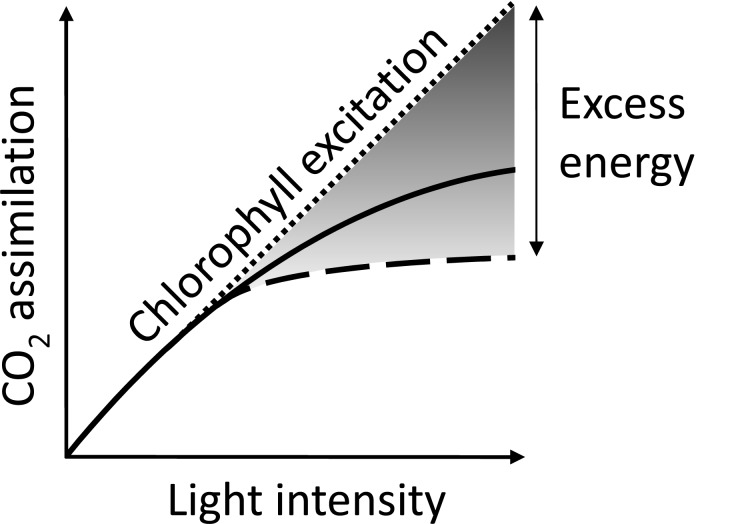
The light response curve of CO2 fixation. With increasing absorbed quanta (dotted line), carbon assimilation eventually saturates and the difference between the two is excess energy. The level when photosynthesis saturates is lowered under unfavourable conditions, as represented by the dashed line.

Natural photosynthesis is a highly regulated process. Several mechanisms help to protect the photosystems against light-induced damage (photoinhibition) when photon flux densities exceed the photosynthetic capacity. Moreover, the intensity when light becomes excess depends on the environment. Hence, in unfavourable conditions light saturation occurs at lower intensities (Fig. **1**). Excess energy that cannot be used to drive photosynthesis enhances the production of reactive oxygen species (ROS) and induces photooxidative damage. Although some regulatory mechanisms may only be important in a living organism, energy dissipation and alternative electron pathways could be relevant for improving the stability of technical devices based on the use of whole photosynthetic organisms like unicellular algae or of isolated photosystems [35]. This review will cover the different levels that regulate photosynthesis in natural systems by using examples from higher plants and the model green alga *Chlamydomonas reinhardtii*. We start by covering the various pathways of light-induced production of ROS, major sources of ROS in plants (for reviews see [36-40]), and then cover how this relates to photoinhibition. The review continues with how excess energy can be dissipated to heat or distributed between the photosystems for protection, but also for influencing the production ratio of ATP:NADPH. Furthermore, we will describe the various electron transport pathways and highlight their importance, from linear to pseudocyclic flow (also called the Mehler reaction) and cyclic, which seems particularly relevant to photoinhibition of PSI. The control of excitation pressure in combination with the partitioning of different electron transport pathways influences the light-dependent formation of ROS, thus is paramount in controlling the stability and longevity of the photosynthetic apparatus. 

## GENERATION OF REACTIVE OXYGEN SPECIES AND PHOTOINHIBITION

**Fig. (2):**
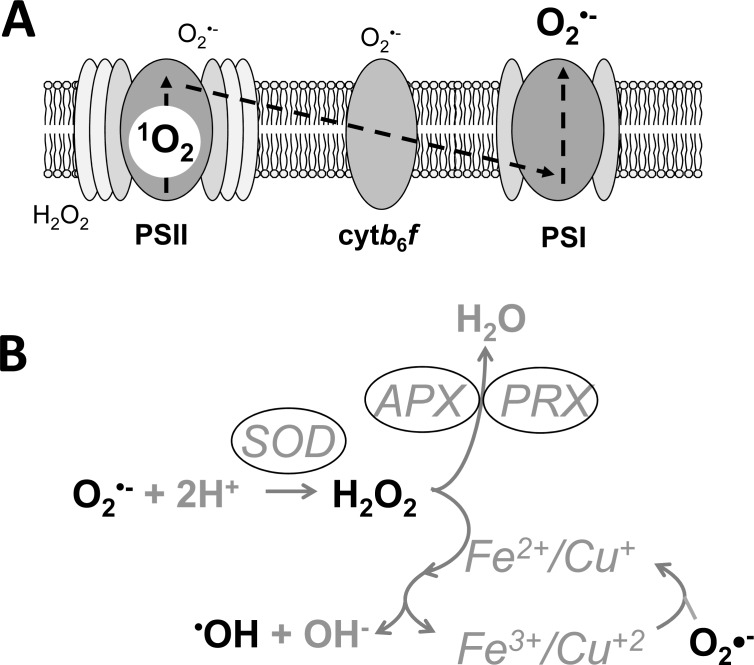
Sites of reactive oxygen species (ROS) production at the thylakoid membrane. **[A]** Singlet oxygen (^1^O_2_) production occurs from charge recombination reactions in photosystem II (PSII), whereas superoxide (O_2_^•-^) is predominantly produced by single electron reductions of O_2_ at the acceptor side of photosystem I (PSI). Other electron carriers, such as cytochrome *b*_6_*f* (Cyt *b*_6_*f*) may also produce negligible amounts of O_2_^•-^ or hydrogen peroxide (H_2_O_2_). The dotted arrows represent electron flow. **[B]** The dismutation of O_2_^•-^ to H_2_O_2_ is catalysed by superoxide dismutase (SOD), which in the chloroplast is broken down by ascorbate peroxidase (APX) and peroxiredoxins (PRX). In contact with Fe^2+^ or Cu^+^, H_2_O_2_ can produce the hydroxyl radical (HO^•^) and Fe^3+^/Cu^2+^ can be recycled with O_2_^•-^.

Excitation of pigments and electron transfer reactions in an oxygen-rich environment inevitably leads to photooxidative damage. This is visible to the eye by a bleaching of the chlorophyll leading to pale green or even whitish leaves under extreme light conditions, especially when plants adapted to shade are suddenly exposed to high light intensities. Light-induced damage of the photosynthetic apparatus is caused by excessive production of ROS such as singlet oxygen (^1^O_2_), superoxide (O_2_^•-^), hydrogen peroxide (H_2_O_2_) and hydroxyl radicals (HO^•^) (Fig. **2**). 

Among the ROS, ^1^O_2_ and HO^•^ are the most reactive species that are able to oxidize lipids, proteins and nucleic acids. Although ROS are important signalling molecules in photosynthetic organisms, high production rates saturate antioxidant defences, lead to oxidative damage and ultimately reduce growth and plant fitness. The up regulation of antioxidant defences is part of an acclimation of photosynthetic organisms to very high light intensities [41]. Moreover, in non-living devices the production of ROS for signalling purposes is unnecessary, allowing ROS production to be minimised. 

^1^O_2_ is produced by the reaction of excited chlorophyll in its triplet state (^3^Chl) with ^3^O_2_ (molecular oxygen is in a triplet state in its ground state). In the reaction centre of PSII, ^3^Chl is generated by charge recombination of the primary radical pair (P680^+^ Phe^-^), with pheophytin (Phe) being the primary electron acceptor and P680 the primary chlorophyll electron donor. When light absorption exceeds the capacity of photosynthetic electron transport, the probability of ^1^O_2_ generation increases. The pathway of charge recombination depends on the energetic of the electron acceptors of PSII (for details see [42, 43]). Charge recombination between the primary quinone electron acceptor (Q_A_) and P680^+^ can proceed via an indirect pathway and the repopulation of the primary radical pair or directly into the ground state of P680. The indirect pathway leads to the formation of ^3^Chl and ^1^O_2_ while the direct pathway is safe (for a more detailed description see [44]). A regulation mechanism has been described by which the yield of ^1^O_2_ production is lowered in PSII with an inactive water-splitting complex. According to this mechanism, ^1^O_2_ generation in PSII is controlled by a regulation of midpoint potential of Q_A_. The yield of ^1^O_2_ formation is lowered when Q_A_ is in its so-called high potential form, i.e., when the midpoint redox potential of Q_A_ is shifted to a more positive value. This shift in the midpoint potential allows a direct recombination of P680^+^Q_A_^-^ to its ground state without repopulating the primary radical pair P680^+^Phe^-^ [42, 44, 45]. PSII centres with high potential Q_A_ have been observed under different physiological conditions *in vivo*: 1) In green algae prior to photoactivation (the light-dependent assembly of the Mn cluster) [46], and 2) in leaves of higher plants under high light conditions [40]. The dependence of the amount of ^1^O_2_ generation in PSII upon the midpoint potential Q_A_ has been demonstrated by electron paramagnetic resonance (EPR) spectroscopy *in vitro* using a spin probe [47] and *in vivo* by a specific fluorescence dye in *Chlamydomonas *[48, 49]. It has been shown that the yield of ^1^O_2_ generation correlates with the loss of the D1 protein, one of the main subunits of the PSII reaction centre [50, 51]. Although chlorophyll-containing light hravesting complex (LHC) of photosystems contain many more chlorophylls, they are less susceptible to damage by ^1^O_2_. In native systems, these antennas are well protected against ^1^O_2_ formation by nearby carotenoids, including xanthophylls, which efficiently quench ^3^Chl [52, 53].

Photoinhibition and degradation of the D1 protein takes place over a large range of light intensities, although a net loss of PSII activity is only observed at high light intensities since the repair of PSII is very efficient *in vivo* [54]. However, at very low light intensities when the secondary PSII quinone electron acceptor (Q_B_) is only semi-reduced, photodamage and D1 loss can also take place. For example, PSII photoinhibition caused by charge recombination reactions and ^1^O_2_ generation has been observed in green algae at very low light intensities [55] and after excitation of PSII in isolated thylakoid membranes by single turnover flashes [56, 57]. It is not only the midpoint potential of the redox couple Q_A_/Q_A_^-^ that influences the probability of the non-radiative pathway of charge recombination, but also the midpoint potential of the redox couple Phe/Phe^-^ [58]. Interestingly, cyanobacteria have two genes for distinct D1 proteins, a main subunit of the PSII reaction centre, with different amino acids at position D1-130. Special D1-E130 proteins are expressed only during high light conditions [59], and are thought to shift the redox potential of Phe to enhance charge recombination via the safe non-radiative pathways, thereby lowering the yield of ^1^O_2_ generation [43]. Similar to the high light isoform of the D1 protein in cyanobacteria, a glutamate occupies the position D1-130 in all higher plants with known sequences. In *Chlamydomonas*, substitution of alanine at D1-251 of the Q_B_ binding pocket to cysteine improved tolerance to cosmic radiation in space-flight and cell survival after returning to earth [60]. PSII can undergo post-translational modifications associated to stress protection and repair. For example, the phosphorylation of the PSII-associated chlorophyll-binding protein CP29 protects from cold-stress [61]. Moreover, the D1 subunit is under a circadian-regulated phosphorylation pattern [62] and requires dephosphorylation before it undergoes degradation [63]. Phosphorylation is also key to thylakoid membrane folding enabling access of repair enzymes [64] and the migration of photosystem antennas in state transitions, as discussed later. For recent detailed reviews on photosynthesis-related phosphorylation readers are directed towards [65] and [66].

Beside ^1^O_2_ other ROS play an important role in light-induced damage of the photosystems. Superoxide is mainly generated at the acceptor side of PSI in the so-called Mehler reaction [67, 68]. In this reaction, O_2_ is reduced by ferredoxin or by the PSI iron-sulphur acceptor F_x_. In algae and cyanobacteria reduction of O_2_ can be the dominant electron transport pathway [69], such as before the light-induced activation of the Calvin-Benson cycle [70], while in higher plants its importance as alternative electron sink is thought to be less important [71, 72]. It has been recently reported that gymnosperms have an increased capacity of the Mehler reaction (about 10 % of the maximum electron flow) compared to angiosperms, but only during dark to light transition before the Calvin cycle is active [73]. In angiosperms it is thought that the importance of the Mehler reaction increases under stress conditions, such as drought, when the CO_2_ availability is limited by stomatal closure [71]. In addition it has been reported that the photoperiod plays a role in the partition between linear electron flow to NADP^+ ^and Mehler reaction [74]. Further investigations are needed to elucidate the physiological importance of the Mehler reaction, if and how it is regulated *in vivo*.

Besides being generated at the acceptor side of PSI, O_2_^•-^ can also be produced *in vitro* at the level of the cytochrome *b*_6_*f* complex (cyt*b*_6_*f*) [75] and at the acceptor side of PSII. In addition, the cytochrome *b*559, an intrinsic protein subunit of PSII can act, depending on its redox potential as an oxygen reductase, as a superoxide reductase or as a superoxide oxidase [76]. However, the capacities of these pathways of O_2_ reduction seem to play only very minor roles in an intact, functional electron transport chain in thylakoid membranes. 

A major source of photosynthesis-associated ROS is the H_2_O_2_ produced by photorespiration during the recycling of bi-products of the oxygenase activity of RubisCO [77]. This occurs outside the chloroplast in the peroxisome and will not be discussed here. However, H_2_O_2_ is generated in isolated chloroplasts, thylakoids and PSI or PSII preparations by the dismutation of O_2_^•-^, either spontaneously or catalyzed by superoxide dismutase (SOD). Small amounts of H_2_O_2_ can also be generated directly by incomplete water splitting at the donor side of PSII as has been shown *in vitro* using isolated PSII membranes [78]. Furthermore, it can be formed by the reduction of O_2_^•-^ by plastoquinol [79]. H_2_O_2_ itself is not that toxic, but in the presence of transition metals, such as Fe^2+^ or Cu^+^, it is converted to the highly reactive HO^•^ radical (Fig. **2**). Apart from the Fenton reaction, HO^•^ may also be formed by the reduction of peroxide by metal centres coordinated to the proteins involved in electron transport.

In the intact chloroplasts, several enzymes are present that detoxify ROS. O_2_^•-^ is dismutated to H_2_O_2_ by SOD containing Cu and Zn (Cu/Zn-SOD) or Fe (Fe-SOD) as a cofactor. H_2_O_2_ is mainly detoxified by ascorbate peroxidase (APX) [80] and by chloroplast-located peroxiredoxins (PRX) [81]. While APX activity requires ascorbate, PRX activity is dependent on re-reduction by thiol or thioredoxins. The ascorbate (20-300 mM) and glutathione (GSH; 0.5 - 3.5 mM) content of the chloroplast [82, 83] is sufficiently high enough to enable a very efficient H_2_O_2_ detoxifying system [69]. Furthermore, thiols and thioredoxins are substrates for glutathione peroxidases and glutathione-s-transferases, which are important in detoxifying reactive lipid species formed by ^1^O_2_ [84, 85]. ^1^O_2_ can be scavenged by tocopherol, plastoquinone, carotenoids and by ascorbate [86-88]. Despite ascorbate, these scavengers are present in isolated systems and will help to protect the photosystems in the light. However, they have limited capacity because regeneration cannot take place and after a given time they become exhausted. The addition of catalase, a non-chloroplast located enzyme, increased the efficiency and stability of thylakoid bio-electrodes [8] confirming that H_2_O_2_ production can be an issue in artificial devices.

It is accepted by the majority of researchers that ROS directly damage photosystems with PSII being more vulnerable against oxidative damage than PSI. However, some researchers have suggested that the repair mechanism of the D1 protein is solely damaged by ROS, and not the PSII reaction centre itself (see Special Issue Physiologia Plantarum 2011 for the current debate). *In vivo*, the repair of PSII is so efficient that damage is only transitory [54]. Addition of methylviologen to isolated thylakoids, which enhances O_2_^•-^ at the acceptor side of PSI, still led to greater inhibition of PSII than PSI [89], showing that PSII is much more susceptible to ROS-induced damage than PSI, even when the site of production is located at PSI. It is intriguing that the D1 reaction centre at the heart of PSII photoinhibiton has remained highly susceptible to photo-damage in all oxygenic photosynthetic organisms and at all light intensities. Despite the apparent wastefulness of PSII photoinhibition, it can also be regarded as a regulatory mechanism of photosynthesis, since it lowers linear electron transport under excess light conditions and may thereby prevent photoinhibition of PSI [90]. Photoinhibition of PSI has a higher impact on the performance of the photosynthetic apparatus since no efficient repair cycle exists [90].

## REGULATION OF LIGHT HARVESTING

What makes photosynthesis remarkable is that it efficiently functions under highly fluctuating photon flux densities, under environmental constraints and in accordance with the metabolic demands of the organism. Photoregulation is coordinated at multiple levels; At the pigment and protein levels via energy-transfer processes involving carotenoids and chlorophylls, at the membrane and cellular levels with supramolecular organization in the thylakoid membrane, at the cellular level by chloroplast relocation and at the organism level including heliotropism of plants and phototaxis of microorganisms. Within the thylakoid membrane, light harvesting complexes facilitate in capturing light energy and its transfer to the reaction centres for charge separation. This partitioning between light harvesting and reaction centres provides an opportunity in regulating how much and to which reaction centre energy is delivered to, thereby preventing excessive excitation, ROS production and the costs associated with photoinhibitory damage. Collectively, this plethora of regulatory mechanisms controlling light energy in intact organism is known as non-photochemical quenching (NPQ), due to their detection by measurements of chlorophyll fluorescence quenching that are distinct from photochemical quenching (i.e., use of the captured light energy in chemical reactions such as CO_2_ fixation). For a complete guide on chlorophyll measurements of photosynthetic efficiency readers are directed to [91]. There are three components to NPQ; 1) dissipation of excess light energy in antenna to heat before it reaches the reaction centre (qE), 2) state transitions where light adsorption is balanced between the photosystems by movement of antenna (qT) and 3) ‘short term’ photoinhibition of PSII (qI), which recovers slower than qE or qT [92, 93]. As discussed below, qT and qE are governed by the plastoquinone (PQ) pool redox state and the thylakoid proton-motive force, respectively, which makes photosynthesis a highly efficient self-regulated process. For a comprehensive review on how photosynthetic organisms respond to excess light see [94].

## THE QE COMPONENT OF NPQ

**Fig. (3):**
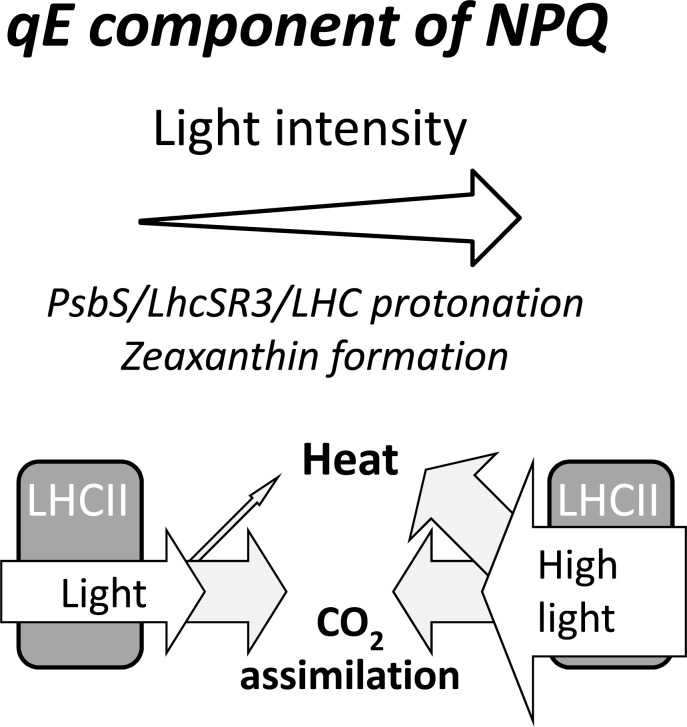
Mitigating excess light via the qE component of non-photochemical quenching. With increasing light intensities, the light-dependent production of a proton gradient across the thylakoid membrane induces two mechanisms that dissipate excess light energy to heat before it reaches the photosystem II reaction centre. Acidification of the lumen 1) enhances the enzymatic conversion of violaxanthin to antheraxanthin and zeaxanthin, and 2) protonates key residues of light harvesting complex II and PsbS (vascular plants) or LhcSR3 (green algae), which together participate in the dissipation of excess energy at the level of the PSII antenna preventing excess light from causing damage.

The qE component of NPQ, where light energy is dissipated before reaching the reaction centres, has been assigned to a synergistic action of the ∆pH since a low pH in the thylakoid lumen activates the xanthophylls cycle and leads to protonation of luminal residues of proteins such as PsbS and LhcSR3 that reduce energy transfer from the antenna to the PSII reaction centre (Fig. **3**). In the xanthophyll cycle, the low pH activates a de-epoxidation of violaxanthin to zeaxanthin and requires minutes to hours to become fully activated [95, 96], but zeaxanthin can temporarily remain active after the loss of the ΔpH [93]. Lutein is another xanthophyll implicated in qE and protection from high light in both *Arabidopsis* and *Chlamydomonas* [52, 97]. Another ∆pH-dependent component of qE is protonation of light harvesting complexes (LHC) and an LHC-type protein, which rapidly induces NPQ within seconds [98]. In higher plants this trans-membrane LHC-type protein is PsbS [99], whereas in green algae (e.g., *Chlamydomonas*) it is LhcSR3 [100]. Differences between the proteins include PsbS being constitutively expressed and not binding pigments, whereas LhcSR3 is highly inducible by excess light and binds chlorophyll and carotenoids [101]. Moreover, the mechanism of LhcSR3 is less dynamic than PsbS and leads to quenching even in low light, which is perhaps why LhcSR3 is a high light-inducible protein [101, 102]. Evidence suggests the switch from LhcSR3 to PsbS happened when organisms colonised the land and was likely associated with the extra stresses associated with terrestrial life [103]. Mosses can contain both proteins as has been shown for *Physcomitrella patens* [104] and *Rhytidium rugusum* (data not shown). Regardless, mutants of *Arabidopsis* or *Chlamydomonas* deficient in either PsbS or LhcSR3, both referred to as *npq4*, have retarded abilities to dissipate excess energy and show sensitivity to high or naturally fluctuating light [100, 105]. The aggregation of LHC II trimers and detachment from PSII is also observed during qE induction [106]. Due to the influence of ΔpH on qE, a regulation of ATP synthase activity, which uses the ΔpH to produce ATP also influences NPQ [107], possibly through thioredoxin-mediated redox control, but this has yet to be confirmed [108]. Recommended introductory reviews to qE are [102, 109] for model plants and [110] in other plants, but also see [52, 111] for further debate. 

We have recently shown *in vitro* using *Arabidopsis* chloroplasts that ^1^O_2_ production is influenced by the presence or absence of PsbS-dependent NPQ capacity [112]. Other markers of ^1^O_2_ production are the oxidation of β-carotene inside the reaction centre and of prenyllipids such as α-tocopherol and plastoquinone [86]. It has also been reported that β-carotene is more oxidised in *Arabidopsis* *npq4* than wild-type following excess light stress, confirming qE protects against reaction centre damage by ^1^O_2_ [113]. In addition to qE, other mechanisms of energy dissipation operate in specialised situations. For example, a light- and nigericin-insensitive activation of the xanthophyll cycle has been shown in a desiccation-tolerant fern and in lichens [114]. Moreover, an induction of non-radiative charge recombinations in PSII (see above) occurs in cyanobacterial desert crusts in response to high light, avoiding the formation of ^3^Chl and associated ^1^O_2_ [115]. Although NPQ is essential to the contribution of desiccation tolerance [116], the contribution of the xanthophyll cycle to NPQ is not always supported, indicating that other mechanisms, such as chlorophyll cations that are very efficient quenchers [117], are responsible for the desiccation-induced NPQ [118]. However, xanthophylls may play other roles in protection from excess light due to their efficient ability to scavenge ROS, such as zeaxanthin protecting from ^1^O_2_ [119, 120] and neoxanthin from O_2_^•-^ [121]. In cyanobacteria, a different type of qE has been described which relies on the light-induced conversion of the Orange-Carotenoid-Protein (OCP) to its active red form that quenches fluorescence (for review see [122]).

## THE QT COMPONENT OF NPQ

**Fig. (4):**
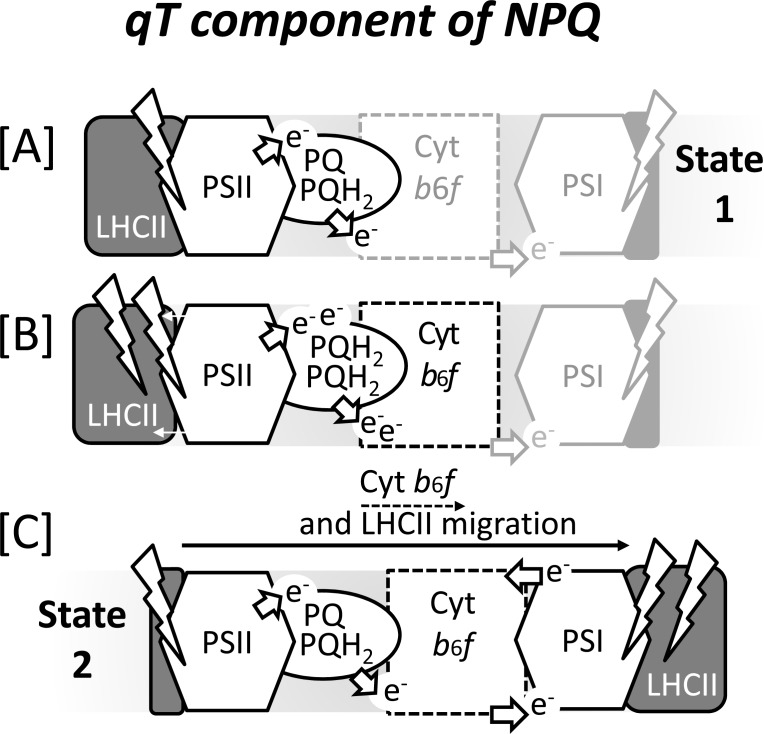
Balancing light absorption by the photosystems via state transitions poised by the redox state of the plastoquinone pool. **[A]** Under moderate light, the redox state of the plastoquinone pool (PQ/PQH_2_) remains largely oxidised allowing linear electron flow, and the majority of light harvesting complex II (LHCII) is at PSII (state 1). **[B]** If PSII becomes over-excited relative to PSI, the PQ pool becomes over-reduced, favouring the binding of PQH_2_ to the Qo site of the cytochrome *b*_6_*f* complex (cyt*b**_6_**f*), which induces a kinase to phosphorylate LHCII and the movable part of LHCII migrates to PSI. **[C]** The migration of LHCII to PSI (state 2) reduces excitation pressure at PSII lowering linear electron transport so that the PQ pool becomes re-oxidised.

Another light-inducible response, the so-called state transitions, influences excitation delivery to the photosystems. Light Harvesting Complex II (LHCII) can migrate to change the energy deliverance to PSII or PSI. Moreover, the movement of LHCII away from PSII relieves excitation pressure [123]. The migration of LHCII is under governance of the plastoquinone pool redox state, and therefore, the relative activities of PSII and PSI. Under high PSII activity and a reduced plastoquinone pool, plastoquinol binds to the Qo site of the cyt*b*_6_*f*, which activates protein kinase STN7 to phosphorylate LHCII [124]. This is a pre-requisite for its movement to PSI and the induction of ‘state 2’ (Fig. **4**). A more oxidised plastoquinone pool leads to the dissociation of plastoquinol from the Qo site of cyt*b_6_f*, thereby deactivating the kinase and activating a phosphatase (as reviewed by [65]). Consequently LHCII migrates back to PSII, referred to as ‘state 1’. State transitions play a minor role in higher plants while they are highly important in green algae. In *Arabidopsis*, where only up to 15-20% of LHCII can be mobilised [124, 125], state transitions are important in acclimating to changing light intensities [126]. However, in *Chlamydomonas* 70- 80% of can disassociates from PSII [127], but only 20 % associates to PSI [127, 165]. It had been accepted in the literature for a long time that in *Chlamydomonas* the transition from state 1 to 2 is accompanied by a switch from linear to cyclic electron flow [128]. However, it has recently been demonstrated that this is not necessarily the case [129]. Although both state transitions and cyclic electron flow are induced by reducing conditions, the migration of LHCII to PSI is not a prerequisite for the induction of cyclic electron flow [129]. 

The activation of high light responses are upregulated at different times and at different intensities depending upon the organism, presumably to address the different demands and adjustments. The fasted responses are qE (first minutes upon exposure to high light, followed by state transitions (qT) and finally leading to photoinhibition of PSII (qI). Although each mechanism has unique functions they also possess overlapping protective roles. 

## REGULATION OF THE ELECTRON TRANSPORT CHAIN

Beside the regulation of the amount and distribution of light energy to the reaction centres, the activity of the electron transport chain can be down regulated at different sites. The size of the ΔpH is the most important component that not only controls qE (see above), but also regulates electron transport at the level of the cyt*b*_6_*f* and at the donor side of PSII. A decreased luminal pH limits electron transport by slowing the activity of the cyt*b*_6_*f*, a regulation mechanism called “photosynthetic control” [130, 131]. The lumen pH in *Arabidopsis* leaves under ambient CO_2_ was estimated to range from approximately pH 7.5 to 6.5 under weak and saturating light, respectively [132]. These moderate pH values in the lumen allow regulation at the antenna level via qE and via electron transport through the cyt*b*_6_*f*, as well as preventing acid-induced damages. The pH value for zeaxanthin accumulation and PsbS protonation was estimated to be about 6.8 [132]. When net ATP synthesis is zero, the pH in the lumen can decrease as low as pH 5.2 (for review see [130], a pH at which the water-splitting activity and the reduction kinetics of P680^+^ start to be slowed down [133]. Below pH 5.5, Ca^2+^, an obligatory co-factor of the water-splitting complex is reversibly removed, evoking a shift of Q_A_ to the high potential form [134] and protecting PSII against ^1^O_2_ generation (see above). 

**Fig. (5):**
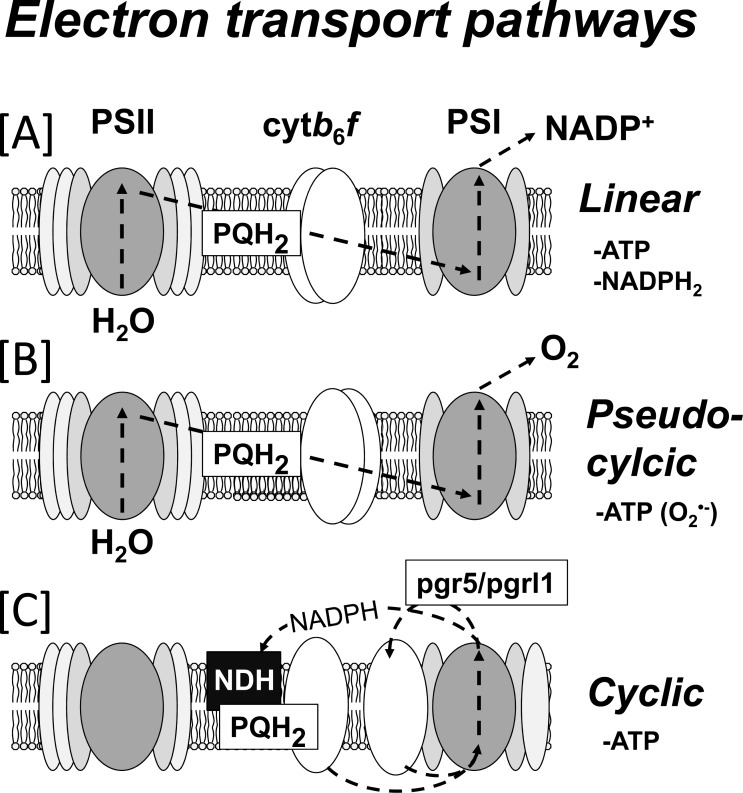
Photosynthetic electron transport pathways. In linear electron flow **[A]** electrons released by water-splitting in photosystem II (PSII) reduce plastoquinone to plastoquinol (PQH_2_), which migrates to the cytochrome *b*_6_*f* complex (cyt*b**_6_**f*). Plastoquinol is oxidized by the cyt*b**_6_**f* and protons are released into the thylakoid lumen. Electrons are transported to plastocyanin (PC), which is the electron donor to photosystem I (PSI). At the acceptor side of PSI electrons are donated to NADP^+^. Alternatively, PSI can reduce O_2_ producing O_2_^•-^ (Mehler reaction or pseudocyclic flow) **[B]**. In cyclic electron flow** [C] **the reducing power from PSI is re-invested back into the electron transport chain via NADPH dehydrogenase (NDH), which reduces the plastoquinone pool, or via a pgr5/pgrl1 protein complex to cyt*b**_6_**f* . All electron flows permit the generation of a thylakoid proton gradient for the generation of ATP, whereas only linear flow produces NADPH/H for carbon assimilation.

Beside processes that are regulated by the luminal pH, alternative pathways of photosynthetic electron transport can release the pressure from the electron transport chain and prevent photoinhibtion. Under conditions of limiting light and no limitation on the electron acceptor side (i.e., sufficient CO_2_), linear electron transport is dominating and electrons released from splitting H_2_O in PSII are used by PSI to reduce electron acceptors such as NADP^+^. As electron acceptors become limited (i.e., low CO_2_ that limits NADPH oxidation for carbon assimilation), pseudocyclic electron flow / Mehler reaction (where O_2_ is the electron acceptor) and cyclic electron flow are increasingly able to compete for reducing power (Fig. **5**). In cyclic electron flow the reducing power is not from PSII, but instead recycled back from PSI into the plastoquinone pool and via cyt*b*_6_*f*. This occurs directly via ferredoxin and other proteins, such as PGR5 [135] and PGRL1 [136, 137], or via NAD(P)H dehydrogenases (NDH) [138]. As the reinvested reducing power of cyclic electron flow passes through the Q-cycle of cyt*b*_6_*f*, the accompanied proton transport from stroma to lumen facilitates the formation of ΔpH, and hence, ATP production. For photosynthetic organisms switching between cyclic and non-cyclic pathways provides a degree of flexibility in the ratio of ATP and NAPDH production to meet metabolic needs [139, 140]. This is particularly important in ATP-expensive photosynthesis, such as CAM plants and in the bundle sheath cells of C_4_ plants, which both require a higher ATP:NADPH ratio for CO_2_ fixation than C_3_ photosynthesis. Furthermore, cyclic flow is enhanced when CO_2_ becomes limiting in both higher plants [141] and *Chlamydomonas*, the latter which has a high ATP demand under CO_2_ limitation because CO_2_-concentrating mechanisms operate at the expense of ATP [142]. Cyclic electron flow is clearly linked to stress, but the exact regulatory mechanism that switches it on is still unknown. A joint PSI-cyt*b*_6_*f* supercomplex for cyclic electron flow has been demonstrated biochemically to be formed in *Chlamydomonas* [129, 143], but not yet in higher plants. The NAD(P)H dehydrogenase (NDH) route in *Chlamydomonas* is achieved by a monomeric protein called Nda2 [144], while in higher plants it is achieved by the NDH complex [145, 146]. Both can operate in the dark with non-photosynthetic supplies of NAD(P)H. In *Arabidopsis*, at least, both the PGR5/PGRL1-dependent and the NDH-dependent cyclic pathways seem to be under redox regulation by thioredoxin m4 [147], while the PSI-cyt*b*_6_*f* supercomplex is Ca^2+^-dependent in *Chlamydomonas* [136]. 

As discussed above, cyclic electron transport is enhanced to support the production of a ΔpH under conditions that limit carbon assimilation, including environmental stress. Thus it allows ATP generation without the net formation of reductants, such as reduced Ferredoxin or NADPH. The exclusion of linear electron flow also limits the Mehler reaction and O_2_^•-^ generation. Moreover, cyclic electron flow protects against photodamage of PSI since it keeps the acceptor side oxidized [90, 112, 135, 148, 149]. 

In addition to partitioning between linear electron flow, cyclic flow and Mehler reaction (Fig. **5**), the plastid terminal oxidase (PTOX), which directly oxidises plastoquinol while reducing O_2_ to H_2_O, has been proposed to act as a safety valve and to avoid photo-oxidative damage [150]. PTOX activity may also enhance the formation of a ΔpH, as demonstrated in marine organisms that survive in iron-depleted waters. Here, the cost of building iron-rich PSI is very high, but organisms can operate with PSII activity alone [151]. In alpine plants the level of PTOX protein is elevated, which may be linked to their ability to tolerate harsh conditions like very high irradiation at low temperatures [150, 152]. In agreement with this, PTOX levels are increased in plants exposed to extreme temperatures [153, 154] or to high salinity [155]. In *Chlamydomas* two isoforms of PTOX exist, PTOX and PTOX2. PTOX2 has been shown to keep the PQ pool oxidized in the dark [156]. However, in other model plants such as *Arabidopsis thaliana*, *Nicotiana tabacum* and *Solanum lycopersicum* grown under standard conditions, the role of PTOX in mature leaves is less clear. Overexpression of PTOX did not protect plants from photoinhibition [157] but actually enhanced it in some circumstances [158, 159]. Recent evidence indicates that PTOX rather modulates the balance between linear and cyclic flow than acting as a safety valve [160] since the capacity of electron flow via PTOX has been measured to be very limited in *S. lycopersicum *leaves [157]. Further investigations are needed to show the importance of PTOX as a safety valve under stress conditions.

In summary, the competition for absorbed quanta by alternative electron flows becomes important when the electron acceptor NADP^+^ is limited. These electron flows include cyclic flow around PSI, the Mehler reaction and PTOX activity. Not only does this enable metabolic adjustment through regulating ATP:NADPH ratios, but also releases reducing pressure off the electron transport chain, lowering incidences of charge recombination and preventing ^1^O_2_ production (see above). Hence, the incorporation of a regulatory mechanism to prevent over-reduction of charge carriers could also be attractive to artificial systems that may suffer damage under high loads. 

## COST-BENEFIT RATIO OF REGULATORY MECHANISMS

An obvious cost of photoregulatory processes is the loss in efficiency in photosynthetic yield. A delay in recovery of NPQ from the residual presence of zeaxanthin after high light treatment [93] may have high costs in carbon assimilation of agricultural plants [161]. In the non-native setting of agriculture that strives for maximum growth rates, the protection afforded by NPQ may well be, at times, too conservative. Therefore, opportunities for genetically manipulating light harvesting mechanisms for optimising yields may exist [162]. However, photoregulation prevents photoinhibition, which itself is costly in energetics and resources for repairing damaged reaction centres as well as in the photosynthesis forgone during repair (reviewed by [163]). Other protective expenses to photosynthetic organisms are the investment in antioxidants, such as ascorbate, that require complex biosynthetic pathways and reductants with other enzymes to recycle their activity. Therefore, the cost-benefit ratio of photoregulation versus photoinhibition is extremely complex, especially considering an ecological setting with unpredictable resource availability. As mentioned above, photoinhibition itself is a regulatory process of biological photosystems that can rapidly repair themselves. This brings into question the perhaps impossible task of increasing the longevity of isolated reaction centres in biomimetic systems, but the incorporation of exogenous antioxidants can help [8]. 

## USE OF REGULATORY MECHANISMS FOR TECHNICAL EXPLOITATION

The question arises as to what we can learn from the regulatory mechanisms of natural photosynthesis for using photosynthetic machinery in technical applications. First, one has to distinguish between technical applications that are either based on exploiting whole organisms as biosensors or using isolated complexes enriched with photosynthetic complexes. Inhibition of the photosynthetic electron transport in whole organisms is easily detectable by monitoring chlorophyll fluorescence and is used to detect heavy metal or herbicide pollution in water. Recent exploits using laser printing have achieved greater contact of electrodes with whole cells [164] or thylakoids [23], increasing efficiency of charge transfer and sensitivity of biosensors to the nM range [23, 25]. In an intact organism all natural regulatory mechanism are present and can be activated depending on the environmental conditions. To allow a long-term use of a biosensor based on intact organisms it has to be ensured that enough nutrients and CO_2_ are available and that waste is removed when intact organisms are embedded into a matrix for the technical application. 

Regarding isolated systems, PSII complexes or PSII-enriched membrane fragments have already been tested as sensors to detect herbicides. Instead of using active PSII, with the highly vulnerable water-splitting complex, it may be interesting to use PSII with an inactivated donor side. Inactivation of the donor side leads to the shift of the midpoint potential of Q_A_ to the high potential form as described above. PSII with high potential Q_A_ is protected against damage by ^1^O_2_. Herbicides bind efficiently to these modified PSII and herbicide binding could be detected by either measuring thermoluminescence or herbicide-induced changes in the decay kinetics of chlorophyll fluorescence. Isolated photosynthetic systems may well be devoid of metabolic control required in whole organisms in responding to changes in the environments like fluctuating light, but in an outside environment they will still be subjected to highly variable conditions of temperature and light intensity, affecting efficiency and performance. Therefore, when isolated thylakoid membranes or isolated PSI preparations will be used in a technical device, it may be useful to add a safety valve in analogy to the PTOX or the Mehler reaction present in the natural system. This safety valve should only operate when PSI (or both photosystems in case of thylakoids) becomes saturated. To design such a safety valve, a better understanding of the regulation of the Mehler reaction and of PTOX are first needed before reasonable suggestion can be made based on physiologically relevant protection mechanisms. As the field of bio-sensors and transducers is only in its infancy, we can expect great advances when technological advances are coupled with an enhanced understanding of photosynthesis and its control.
